# The Influence of Exercise Training on Quality of Life and Psychosocial Functioning in Children with Congenital Heart Disease: A Review of Intervention Studies

**DOI:** 10.3390/sports5010013

**Published:** 2017-02-10

**Authors:** Karolijn Dulfer, Willem A. Helbing, Elisabeth M. W. J. Utens

**Affiliations:** 1 Department of Child and Adolescent Psychiatry/Psychology, Erasmus Medical Center Sophia Children’s Hospital, 3015CN Rotterdam, The Netherlands; k.dulfer@erasmusmc.nl; 2 Department of Paediatrics, Division of Cardiology, Erasmus Medical Center Sophia Children’s Hospital, 3015CN Rotterdam, The Netherlands; w.helbing@erasmusmc.nl; 3 Research Institute of Child Development and Education, University of Amsterdam/de Bascule, Academic Center for Child and Adolescent Psychiatry, 1105AZ Amsterdam, The Netherlands; l.utens@debascule.com

**Keywords:** congenital heart disease, children, exercise, sports, quality of life, psychology

## Abstract

Children and adolescents operated upon for congenital heart disease may show reduced exercise capacity and physical activity, associated with lowered quality of life. This review presents intervention studies on the influence of an exercise program on quality of life and psychosocial functioning in children with severe congenital heart disease. Participation in an exercise program among young people with complex congenital heart disease seemed to have positive effects on quality of life and passive leisure time spent. However, more effects of the exercise programs may have been expected. For future research it is important to critically evaluate the content of the exercise programs.

## 1. Introduction

Over the last decades, improvements in diagnostic techniques and pre-, peri-, and post-operative care have resulted in better cardiac outcomes in children with congenital heart disease. In the initial treatment era, more than 80% of children with congenital heart disease died, whereas nowadays more than 90% reach adulthood [1]. 

Children with mild congenital heart disease usually have few physical problems during daily activity. However, children with complex congenital heart disease may experience long-term morbidity, which may be unavoidable even after their heart surgery [2,3]. Compared with healthy peers, these children may have a reduced exercise capacity, which has been associated with a reduced health-related quality of life (HRQoL) [4]. In a recent study, sports participation, especially competitive sports, was related to better self-reported physical quality of life, and, to a lesser extent, to better psychosocial quality of life in children and young adults with congenital heart disease [5]. 

Being less physically active in adolescence may be associated with an increased risk of cardiovascular disease in adulthood [6]. In general, adolescents who are less physically active will be less physically active in adulthood [7]. Therefore, it is important to stimulate children and adolescents with congenital heart disease to participate in exercise to make sure they will remain physically active in the long term. Two systematic reviews [8,9] on the effects of exercise training on physical functioning and exercise capacity have shown that exercise training can be considered an effective method of improving exercise capacity. However, until now, a review specifically focusing on the effects of an exercise program on quality of life and psychosocial functioning in children with complex congenital heart disease has never been published before. 

## 2. Search Strategy

The following electronic databases were searched for English articles, published between January 2010 and December 2016: EMBASE, PUBMED. From the retrieved articles, references were checked for additional articles fulfilling the inclusion criteria. The following search terms were used: “congenital heart disease”, “children” and/or “adolescents”, “exercise” and/or “sports”, and “quality of life” and/or “psychology”. They were entered as Emtree/Medical Subject Headings (MeSH) terms and as title-abstract terms. The abstracts of relevant articles were screened on the basis of the following inclusion criteria: the study design should be interventional (prospective or randomized controlled), the intervention had to consist of any type of exercise or physical activity, the study population should (partly) comprise children/adolescents with a congenital heart disease <18 years old, and the outcome variable should be part of a psychosocial construct or quality of life. 

Abstracts from congresses and/or unpublished data were not included. 

## 3. Study Design and Participants

In total, eight studies were eligible for this review, shown in Table 1. Two of the studies used a randomized, controlled design [10,11], two studies used a prospective, non-randomized design with controls [12,13] and four studies used a prospective design without control groups [14,15,16,17], two of which were pilot studies [16,17]. Most studies included children with severe congenital heart disease, e.g., tetralogy of Fallot, Fontan circulation, transposition of the great arteries [10,13,14,15,16]. Two studies included various diagnoses (both mild and severe) [11,12] and one study included children with pulmonary arterial hypertension [17]. The age range in the all studies was eight to 17 years.

Five studies used a standardized exercise program that combined aerobic exercise training with light static, resistance exercises [10,12,13,16,17], shown in Table 2. Three studies had a set training intensity based on heart rate and were supervised by a physiotherapist [10,12,13]. Two studies used a sports camp in which children could choose in which dynamic sports they would participate [14,15]. One study encouraged children to participate in as much high-intensity short-term activities in daily life as possible [11].

## 4. The Effect of an Exercise Program on Quality of Life

A total of seven studies assessed quality of life as an outcome of exercise training [10,11,13,14,15,16,17]. In one of the two randomized, controlled trials [10], participation in a 12-week standardized aerobic exercise program, under the supervision of a child physiotherapist near their home, improved self-reported cognitive functioning in children with severe congenital heart disease between 10 and 15 years old. They reported to experience less trouble with concentration and learning at school. Their parents reported that their children improved in regards to social functioning; for example, their children played with other children more frequently. Children and adolescents in the intervention group with low quality of life at baseline before exercise training improved in cognitive functioning, motor functioning and pain and physical symptoms, whereas control children with low baseline quality of life, who only received medical care as usual, did not. As to young people between 16 and 25 years old, the exercise program had no effect on their quality of life [10]. 

These short-term improvements of a relatively short-run exercise program are in contrast with the results of a second randomized, controlled trial in a group of children with various diagnoses between 13 and 16 years old [11]. A 52-week eHealth intervention had no effect on self-reported generic and disease-specific quality of life or in influencing daily physical activity in the long term. However, the intervention did not comprise a standardized exercise program but a mobile e-health application to promote physical activity in which adolescents could register their short-term, high-intensity activities of at least 10 min. This may also explain the contrast in results, since in a standardized exercise program [10] the children had to exercise in a gym under the supervision of a physiotherapist and wearing a heart rate monitor to help them perform their exercises within the predetermined submaximal heart rate range. This guaranteed treatment integrity whereas in the eHealth intervention, children had to register their own activities without subjective monitoring or supervision of integrity. The adherence in the standardized exercise program [10,20] was 89%, which means that, on average, four training sessions in 12 weeks were missed. Besides, three participants (3%) dropped out. In the eHealth intervention [11], 35 randomized participants (43%) did not adhere to the eHealth application; 10% of the randomized participants were active users. 

The remaining five non-randomized studies used small samples. Besides, one had a voluntary control group that did not want to participate in the exercise program [13] and four of them did not have any control group [14,15,16,17]. The exercise programs in these five non-randomized studies were very diverse and it was not always clear whether they were standardized; they either used a three-day sports camp without a predetermined training intensity [14,15], a 12-week rehabilitation program in a satellite clinic of a larger hospital or in a center near the child’s home [13], or a 16-week exercise program at home [16]. All studies found promising results regarding quality of life; however, no unambiguous picture can be given. 

## 5. The Influence of an Exercise Program on Psychosocial Functioning

Two studies reported psychosocial functioning as an outcome after an exercise program. Both studies had comparable sample sizes in both the intervention and the control group. However, the first study [12] did not have a randomized, controlled design, and the control group consisted of volunteer control children with congenital heart disease. The other study [18] used a randomized, controlled trial design. The exercise programs differed between the two studies. The first study, with a 30% drop-out rate, used two different interventions in which children participated two times a week: either in a two-week program at a rehabilitation center or in a five-month program in a facility near the child’s home. The second study, with a 3% drop-out rate, used a 12-week program in which the children participated three times a week in aerobic dynamic training in a facility near their home. 

In both studies, the exercise program had no effect on self-reported general anxiety or depressive symptoms. Fredriksen et al. [12] reported a decrease in parent-reported internalizing problems in the intervention group only. Adolescents within the exercise group in the second study [18] reported a decrease in anxiety regarding exercise after the program, whereas those within the control group did not. Remarkably, in this study both adolescents and their parents in the control group reported an improvement regarding internalizing problems, such as anxiety and depression. Possibly, the two comprehensive medical and psychological assessments may have given them a feeling of safety and reassurance. These assessments, combined with the knowledge that they did not have to exercise three times a week, may have been a relief for them, and therefore may have caused the decrease in anxiety and depressive feelings.

In this last study, the exercise program had an effect on self-reported passive leisure time spent [19]. After the program, adolescents spent less time on using the computer. However, this time was not filled with active leisure activities such as walking, cycling or sports activities. This finding could be related to the finding that the exercise program had no effect on sports enjoyment in adolescents in the exercise group. 

## 6. The Influence of Parental Variables on the Effects of an Exercise Program

Since parental variables may influence the well-being of children with congenital heart disease, it is important to identify these parental moderators, especially since parental anxiety and overprotection may hamper the participation of children with congenital heart disease in physical activities [21,22]. One study reported the influence of parental variables on the effects of the exercise training [23]. In that study, adolescents between 10 and 15 years old, whose parents reported anxiety or depressive symptoms, reported a decrease in their social functioning after the exercise program. They indicated that they could play or talk less comfortably with other children. Alternately, they indicated that they felt less at ease with other children.

## 7. Discussion and Conclusion

Although the design and results of the included studies were very diverse, participation in an exercise program among young people with complex congenital heart disease seemed to have positive effects on quality of life and passive leisure time spent. Unfortunately there is no strong evidence emerging from the included studies as to which exercise program had the most effective content to influence quality of life and psychosocial functioning. The only randomized, controlled trial performed until now, with a standardized 12-week exercise program in which children participated three times a week (with 89% adherence) in aerobic dynamic exercise training, reported improvements in quality of life. However, more effects of the exercise programs might have been expected. For future research, it is therefore important to critically evaluate the content of the exercise programs. Most included studies used aerobic exercise training with light static resistance exercises; however, the effects of high-intensity resistance training are unknown. Besides, the programs should be attuned to the individual needs of these young people with complex congenital heart disease to make them more attractive for them and to improve adherence. The use of technological interventions such as virtual reality might motivate children and adolescents to participate in physical activity. A contemporary example is the popularity of Pokémon Go and its effect on physical activity in children and adolescents [24]. Although the relationship between physical activity and quality of life in children with congenital heart disease is not clear [4], such an intervention might improve their quality of life. 

Adding a psychosocial component to the exercise programs could have had a greater impact on quality of life and psychosocial functioning. Morrison et al. designed an exercise program for adolescents with congenital heart disease with psychological methods such as a motivational interview and monthly contact moments to check on the progress of their exercise plan [25]. This intervention improved moderate-to-vigorous physical activity in the intervention group. In addition, although not mentioned, such an intervention could have had an impact on quality of life and psychosocial outcomes. On the other hand, Klausen et al. found that a 52-week Internet-, mobile app–, and SMS-based eHealth program to encourage physical activity improved daily physical activity [11]. However, they found no improvements in generic or disease-specific HRQoL in children compared with control children. The influence of an eHealth motivational program in combination with an aerobic exercise program (monitored on integrity and adherence) on quality of life in children and adolescents with congenital heart disease has, to our knowledge, never been studied in children and adolescents with congenital heart disease.

## 8. Clinical Implications

Comparable to the well-known guidelines for young people in the general population, young people with congenital heart disease should also be encouraged to participate in 60 min of daily physical activity [26]. It is important to especially encourage those children and adolescents with a low quality of life, since they benefit the most from a standardized exercise program. As for the recommendation for clinical practice, in order to identify these children with low quality of life, semi-structured questions regarding sports participation, anxiety towards sports, and depressive symptoms in both children and their parents could be integrated in outpatient visits. This review reported contrasting outcomes as to which exercise (programs) children with congenital heart disease may benefit the most from. For future research, it is recommended to assess the effects of different kinds of exercise programs (e.g., high-intensity resistance training, augmented virtual reality, or adding a psychological, motivational component) in systematically tested, large, multicenter trials to enhance generalizability. Besides, children and adolescents should be involved in designing an exercise program in order to improve their quality of life.

## Figures and Tables

**Table 1:** Studies regarding the influence of exercise training on quality of life in children with congenital heart disease.

Author	Design and Sample (Type of Congenital Heart Disease, and Age Range)	Outcome Measures	Intervention and Procedure	Results
Fredriksen, 2000 [12]	Non-randomized prospective study; intervention n = 55 and controls n = 38 (Surgically operated, various diagnoses *, 10–16 years)	*Emotional and behavioral problems*; YSR (c) CBCL (p)	T1 = baseline T2 = after intervention: either two weeks at rehabilitation facility or over five months, twice a week facility near home	From T1 to T2. Intervention group parents reported regarding their child: - Less externalizing and social problems - Less internalizing problems Control group parents reported regarding their child: - Less externalizing and social problems Child self-reports both groups: no effects
Rhodes, 2006 [13]	Non-randomized prospective study; Intervention n = 15 and controls n = 18 (mostly Fontan circulation, 8–17 years)	*Quality of life*; CHQ-CF87 (c) CHQ-PF50 (p)	T1 = baseline T2 = after a 12-week exercise program, twice a week. T3 = one year after T1	From T1 to T3. Intervention group self-reports: emotional, behavioral, and physical domains improved (not significantly) Control group self-reports: no improvements Parent-reported quality of life outcomes regarding their child were not reported
Moons, 2006a [14]	Prospective study; n = 16 (mostly single ventricle physiology, 10–14 years)	*Quality of life*; CHQ-CF87 (c)	T1 = baseline; start sports camp T2 = after three-day multi-sports camp	From T1 to T2. Self-reported improvements on physical functioning, emotional role functioning, behavioral role functioning, general behavior, and mental health.
Moons, 2006b [15]	Prospective study; n = 25 (mostly single ventricle, Tetralogy of Fallot, Transposition of the Great Arteries, 10–15 years).	*Quality of life*; CHQ-CF87 (c)	T1 = baseline; start sports camp T2 = after three-day multi-sports camp T3 = three months after T1	From T1 to T2. Self-reported improvements on physical functioning, role functioning due to physical problems and due to emotional problems, general health, self-esteem, mental health, general behavior. At T3, sustained improvements on physical functioning and role functioning due to emotional problems.
Dulfer, 2014 [10,18,19]	Randomized controlled trial; intervention n = 54 and controls n = 37 (Fontan circulation or Tetralogy of Fallot, 10–25 years).	*Quality of life*: Tacqol-CF (c) Tacqol-PF (p) TAAQOL-CHD (c) # *Emotional and behavioral problems:* YSR (c) CBCL (p) Anxiety thermometer	T1 = baseline T2 = after a 12-week, three-times-a-week groupwise exercise program supervised by a child physiotherapist.	From T1 to T2. Self-reported improvements in cognitive functioning in 10–15 year olds. Parent-reported improvements in social functioning in 10–15 year olds. Young people between 16 and 25 did not report changes in their quality of life.
Jacobsen, 2016 [16]	Pilot prospective study; n = 14 (Fontan circulation, 8–12 years)	*Quality of life*: PedsQL CF (c) PedsQL PF (p)	T1 = baseline T2 = after a 12-week home-based exercise program, three to four times a week	From T1 to T2. No self-reported improvements in quality of life. Parent-reported improvements in their child regarding overall quality of life, physical, social, school, and psychosocial domains.
Zoller, 2016 [17]	Pilot prospective study; n = 9 (Pulmonary arterial hypertension, mean age 15.2, SD 3.8)	*Quality of life:* Short Form-12 item (c + p)	T1 = baseline T2 = after a 16-week home-based exercise program, two times a week	From T1 to T2. No self-reported or parent-reported improvements on the SF-12 Physical and Mental Component Summary Score.
Klausen, 2016 [11]	Randomized controlled trial; intervention n = 81 and controls n = 77 (Various diagnoses **, 13–16 years)	*Quality of life:* PedsQL-CF generic (c) le PedsQL-CF disease-specific # (c)	T1 = baseline T2 = after a 52-week Internet-, mobile app- and SMS-based program to encourage physical activity	From T1 to T2. An eHealth intervention had no significant effect on generic or disease-specific HRQoL in children compared with control children.

Notes: * Various diagnoses included: transposition of the great arteries, ventricle/atrial septum defect, left/right ventricular outflow tract obstruction, tetralogy of Fallot, functionally univentricular hearts. ** Various diagnoses included: coarctation of the aorta, transposition of the great arteries, Steno-Fallot tetralogy, double outlet right ventricle, truncus arteriosus, atrioventricular septal defect, total cavopulmonary connection surgery. # = disease-specific questionnaire. (c) = self-report, (p) = parent report. CBCL = Child Behavior Checklist, CHQ-CF87 = Child Health Questionnaire Child Form, CHQ-PF50 = Child Health Questionnaire Parent Form, PedsQL-CF = Pediatric Quality of Life Inventory Child Form, PedsQL-PF = Pediatric Quality of Life Inventory Parent Form, SD = standard deviation, TAAQOL-CHD = Congenital Heart Disease-TNO/AZL Adult Quality of Life, TACQOL-CF = TNO-AZL Child Quality of Life Questionnaire Child Form, TACQOL-PF = TNO-AZL Child Quality of Life Questionnaire Parent Form, YSR = Youth Self Report.

**Table 2:** Duration, intensity, adherence, and type of the exercise programs.

Reference	Drop-Out ^1^	Duration (Weeks)	Sessions per Week	Training Intensity	Adherence to Protocol ^2^	Type of Exercise	Training Site/Supervision
Fredriksen, 2000 [12]	36%	2 or 20*	2	65%–80% PeakHR	-	Swimming, football, volleyball, and activities for strength, balance, coordination and flexibility.	Supervised by physiotherapist (HR monitored)
Rhodes, 2006 [13]	16%	12	2 × 60 min	HR at Vth	75%	Aerobic and light weight/resistance exercises	At rehabilitation center or center nearby home. Supervised by physiotherapist
Moons, 2006 [14]	6%	1	3 days	Not mentioned	Not mentioned	Athletics, tennis, baseball, and hockey	Sports camp supervised by sport teachers
Moons, 2006 [15]	36%	1	3 days	Not mentioned	Not mentioned	Soccer, tennis, table tennis, basketball, indoor soccer, judo, trampoline, hockey, handball, volleyball, badminton and baseball	Sports camp supervised by sport teachers
Dulfer, 2014 [10,18,19]	3%	12	3 × 60 min	Submax. HR range **	89%	Aerobic dynamic cardiovascular training	Center nearby home. Supervised by physiotherapist
Jacobsen, 2016 [16]	7%	12	3–4 × 45 min	Not mentioned	Not mentioned	Home exercise routine of dynamic and static exercises	Home based with three in-person exercise sessions.
Zoller, 2016 [17]	-	16	4 × 25 min	60%–70% PeakHR	Not mentioned	Home exercise: bicycle ergometer and theraband muscle tone activities	Home based
Klausen, 2016 [11]	30%	52	Daily 60 min	Not mentioned	57%	Short-term activities of at least 10 min on high intensity	Mobile e-health application

Notes: HR: heart rate; Vth: ventilatory threshold; METS: metabolic equivalent taks; DT: dyspnea threshold; RPE: rating perceived exertion; PeakVO2: ventilatory oxygen peak; b/min: beats per minute and PeakWL: workload peak., 1: percentage of all participants; 2: percentage of fulfilled sessions/total number of (training) sessions, *: Two weeks training in rehabilitation center or 20 weeks training in center nearby home, ** Submax HR Range: resting HR plus 60% to 70% of the HR reserve.
